# A compendium of multi-omic sequence information from the Saanich Inlet water column

**DOI:** 10.1038/sdata.2017.160

**Published:** 2017-10-31

**Authors:** Alyse K. Hawley, Mónica Torres-Beltrán, Elena Zaikova, David A. Walsh, Andreas Mueller, Melanie Scofield, Sam Kheirandish, Chris Payne, Larysa Pakhomova, Maya Bhatia, Olena Shevchuk, Esther A. Gies, Diane Fairley, Stephanie A. Malfatti, Angela D. Norbeck, Heather M. Brewer, Ljiljana Pasa-Tolic, Tijana Glavina del Rio, Curtis A. Suttle, Susannah Tringe, Steven J. Hallam

**Affiliations:** 1 Department of Microbiology and Immunology, University of British Columbia, Vancouver, British Columbia, Canada V63 1Z3; 2 Department of Biology, Georgetown University, Washington, District Of Columbia 20057, USA; 3 Department of Biology, Concordia University, Montreal, Quebec, Canada H4B 1R6; 4 Earth, Ocean and Atmospheric Sciences, University of British Columbia, Vancouver, British Columbia, Canada V6T 1Z4; 5 Department of Civil Engineering, University of British Columbia, Vancouver, British Columbia, Canada V6T 1Z4; 6 Department of Energy Joint Genome Institute, Walnut Creek, California 94598, USA; 7 Biological and Computational Sciences Division, Pacific Northwest National Laboratory, Richland, Washington 99352, USA; 8 Department of Botany, University of British Columbia, Vancouver, British Columbia, Canada V6T 1Z4; 9 Peter Wall Institute for Advanced Studies, University of British Columbia, Canada V6T 1Z2; 10 Genome Science and Technology Program, University of British Columbia, Vancouver, British Columbia, Canada V6T 1Z3; 11 Graduate Program in Bioinformatics, University of British Columbia, Vancouver, British Columbia, Canada V6T 1Z3; 12 ECOSCOPE Training Program, University of British Columbia, Vancouver, British Columbia, Canada V6T 1Z3

**Keywords:** Water microbiology, Microbial ecology, Metagenomics, Proteomic analysis, Sequencing

## Abstract

Marine oxygen minimum zones (OMZs) are widespread regions of the ocean that are currently expanding due to global warming. While inhospitable to most metazoans, OMZs are hotspots for microbial mediated biogeochemical cycling of carbon, nitrogen and sulphur, contributing disproportionately to marine nitrogen loss and climate active trace gas production. Our current understanding of microbial community responses to OMZ expansion is limited by a lack of time-resolved data sets linking multi-omic sequence information (DNA, RNA, protein) to geochemical parameters and process rates. Here, we present six years of time-resolved multi-omic observations in Saanich Inlet, a seasonally anoxic fjord on the coast of Vancouver Island, British Columbia, Canada that undergoes recurring changes in water column oxygenation status. This compendium provides a unique multi-omic framework for studying microbial community responses to ocean deoxygenation along defined geochemical gradients in OMZ waters.

## Background & Summary

Marine oxygen minimum zones (OMZs), areas of low dissolved oxygen (O_2_) in subsurface waters, result from a combination of microbial respiration of organic matter raining down from surface waters and increased water column stratification^1–3^. As O_2_ becomes limiting, microbial communities shift their energy metabolism to use alternative terminal electron receptors in a thermodynamically defined order resulting in increased nitrogen loss and the production of climate active trace gases including nitrous oxide (N_2_O) and methane (CH_4_)^4–9^. Currently, OMZs are expanding throughout the global ocean^3,10–14^ making it increasingly important to define the microbial metabolic networks driving nutrient and energy cycling under changing levels of water column O_2_-deficiency^2,10,15^.

Advances in sequencing technology are enabling the study of microbial communities at unprecedented scales^16^. Tag sequencing uses primers to amplify specific target genes and subsequently sequence them on high-throughput platforms, generating molecular barcodes that can be used to study microbial community structure and function depending on the marker. The small subunit ribosomal RNA (SSU rRNA) gene is a universally conserved marker commonly used to compare microbial communities among and between samples in a quantitative manner^17^. Metagenomic sequencing enables reconstruction of microbial community metabolic potential at the level of genes and pathways over relatively long time scales reflecting persistent information storage in the environment. However, metabolic potential does not necessarily indicate active processes as different genes may be expressed under changing environmental conditions^18^. Metatranscriptomic sequencing opens a window into microbial community gene expression patterns on relatively short time scales reflecting environmental response patterns^18^. Similarly, metaproteomics identifies proteins present in the microbial community on intermediate times scales, providing an alternative perspective on post-translational regulation and catalysis^18^. Collectively, multi-omic methods (DNA, RNA and protein) can be used to chart microbial metabolism at individual, population and community levels along defined geochemical gradients in OMZs^19–26^.

Saanich Inlet, a seasonally anoxic fjord on the coast of Vancouver Island, British Columbia is a model ecosystem for studying microbial community responses to changing levels of water column O_2_-deficiency^8,20–22,27–33^. As microbial communities shift their energy metabolism to use alternative electron acceptors within the Saanich Inlet water column, differential modes of metabolic coupling can be observed including a modular denitrification pathway coupled to sulphide oxidation^20,21,28^. Such metabolic coupling is reminiscent of symbiotic associations and is likely widespread in OMZ ecosystems^2^. Although current research efforts are increasingly focused on defining co-metabolic innovations among and between ubiquitous OMZ microorganisms, many open questions remain regarding the regulatory and ecological dynamics modulating microbial community responses to OMZ expansion^2,5,20–22,34^.

**Figure 1:**
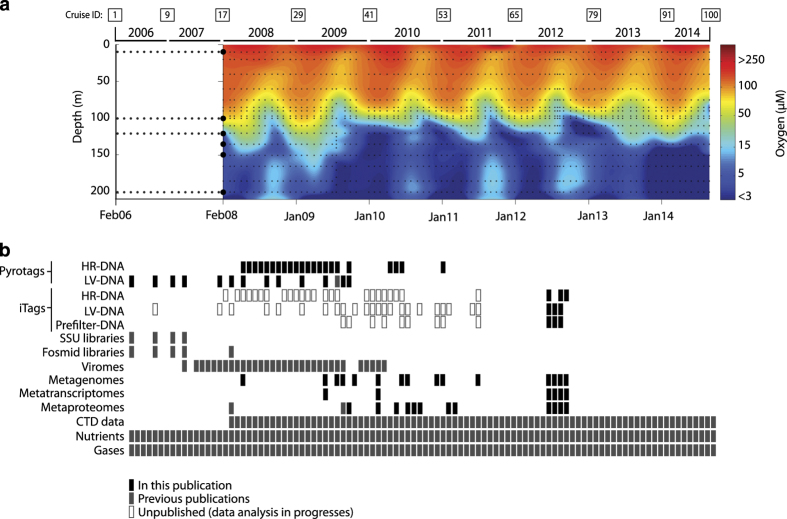
Summary of multi-omic samples collected in Saanich Inlet time series. (**a**) Oxygen concentration contour for CTD data (February 2008 onward)^35^ indicating 16 sampling depths for water column geochemistry and high-resolution (HR) DNA samples for SSU libraries (small black dots) and six major depths for large volume (LV) samples for meta-genomics, -transcriptomics, -proteomics and LV SSU libraries (large black dots). (**b**) Sample inventory from February 2006 to October 2014 indicating multi-omic datasets included in this manuscript (solid black), in previous publications (gray) and accompanying datasets currently undergoing processing and analysis (open gray).

Here we present a compendium of multi-omic sequence information from the Saanich Inlet water column (Fig. 1) encompassing 412 SSU rRNA pyrotag (V6-V8 region) samples (Data Citation 1), 82 SSU rRNA iTag (V4 region) samples (Data Citation 1) (Table 1), 90 metagenomes (Data Citation 1) (totalling 4.1 TB of cleaned reads or 16.2 GB of assembled data), 62 metatranscriptomes (Data Citation 1) (including 46 unique samples and 16 replicates, totalling 1.7 TB of cleaned reads or 2.88 GB of assembled data) and 68 metaproteomes (64 unique samples, totalling 5.2 million unique proteins) (Data Citation 2) (Table 2). Together sequence read data is approximately 5.9 TB of data, comparable to nearly 2,000 human genome equivalents. These data sets, in combination with a cognate geochemical compendium^35^ comprise a unique time-resolved framework for reconstructing microbial community metabolism along defined geochemical gradients and promoting the development of models to predict microbial community responses to changing levels of water column oxygen-deficiency^11,22,36^.

## Methods

### Environmental sampling

Time-series monitoring in Saanich Inlet was conducted on a monthly basis aboard the *MSV John Strickland* at station S3 (48°35.500 N, 123°30.300 W) as described in ref. 8. Samples for large volume (LV) SSU rRNA gene tags, metagenomics, metatranscriptomics, and metaproteomics were taken from six major depths spanning the oxycline (10, 100, 120, 135, 150, and 200 m). Samples for high-resolution (HR) SSU rRNA gene tag sequencing were taken at 16 depths along the oxycline (10, 20, 40, 60, 75, 80, 90, 97, 100, 110, 120, 135, 150, 165, 185 and 200 meters). A detailed seawater sampling video protocol can be found online at http://www.jove.com/video/1159/seawater-sampling-and-collection^37^.

During sampling procedure, large volume waters were collected in 2×12 l Go-Flow bottles on a wire separated by less than one meter. Waters for metatranscriptomics and metaproteomics (2 l each) were collected consecutively from the Go-Flow into 2 l Nalgene bottles with sterile silicon tubing immediately following sampling for dissolved gases to minimise changes in microbial gene expression. Waters for metatranscriptomics were filtered on-board within 8 min of collection on deck, followed immediately by filtering waters for metaproteomics. For both metatranscriptomics and metaproteomics, a peristaltic pump was used to filter waters through a 0.22 μm Sterivex filter with an in-line 2.7 μm GDF pre-filter. Following removal of residual seawater by extrusion using a 10 cc or 60 cc syringe, 1.8 ml of RNAlater (Ambion) was added to metatranscriptomic sample filters and 1.8 ml of sucrose lysis buffer was added to metaproteomic sample filters. Filters were placed on dry ice, returned to lab and stored at −80 °C until processing. A detailed small volume filtration protocol can be found online at http://www.jove.com/video/1163/small-volume-1-3l-filtration-of-coastal-seawater-samples^37^.

Waters for LV SSU rRNA gene tags and metagenomics were combined from two Go-Flows into a 20 l carboy using sterile silicon tubing. Carboys were then transported back to the lab for filtration approximately 6–10 h after collection. Approximately 10 l was filtered through 2 Sterivex filters per depth as described above. Following removal of residual seawater by extrusion, 1.8 ml of sucrose lysis buffer was added to LV sample filters. Filters were placed on dry ice, returned to lab and stored at −80 °C until processing. A detailed large volume filtration protocol can be found online at http://www.jove.com/video/1161/large-volume-20l-filtration-of-coastal-seawater-samples^38^.

Waters for high resolution (HR) SSU rRNA gene tags were collected in 12 l Go-Flow or 8 l Niskin bottles and transferred into 1 l (February 2006—September 2009) or 2 l (September 2009 onward) Nalgene bottle with sterile silicon tubing and stored on ice. Bottles were then transported back to the lab for filtration approximately 12–16 h after collection. A peristaltic pump was used to filter waters through a 0.22 μm Sterivex filter without a pre-filter. Following removal of residual seawater by extrusion, 1.8 ml of sucrose lysis buffer was added to HV sample filters. Filters were placed on dry ice, returned to lab and stored at −80 °C until processing. A detailed small volume filtration protocol can be found online at http://www.jove.com/video/1163/small-volume-1-3l-filtration-of-coastal-seawater-samples^37^.

### Environmental DNA, RNA and protein extraction

Genomic DNA (HR and LV) was extracted from Sterivex filters as described in^27^. Video protocols describing the extraction process in detail can be found online at http://www.jove.com/video/1352/dna-extraction-from-022-m-sterivex-filters-cesium-chloride-density^39^. Briefly, after thawing Sterivex filters on ice, lysozyme (Sigma) was added and incubated at 37 °C for 1 h with rotation followed by addition of Proteinase K (Sigma) and 20% SDS and incubation at 55 °C for 2 h with rotation. Lysate was removed by extrusion and then filters were rinsed with sucrose lysis buffer. Combined lysate was extracted with phenol:chloroform followed by chloroform and the aqueous layer collected and loaded onto a 10 K Amicon filter (Millipore) cartridge, washed three times with TE buffer (pH 8.0) and concentrated to a final volume between 150–400 μl by centrifugation.

Total RNA was extracted from Sterivex filters using the mirVana miRNA Isolation kit (Ambion)^19,25^ modified for Sterivex filters. Briefly, after thawing filters on ice, RNAlater was removed by extrusion, followed by rinsing with Ringer’s solution (Sigma) and incubation at room temperature for 20 min with rotation. Ringer’s solution was removed by extrusion, followed by addition of lysozyme and incubation at 37 °C for 30 min with rotation. Lysate was removed by extrusion into 15 ml tube and subjected to organic extraction as described in the mirVana kit protocol, adjusting for total volume of lysate. Removal of DNA and purification of total RNA were conducted using the TURBO DNA-free kit (Thermofisher) and the RNeasy MinElute Cleanup kit (Qiagen) protocols respectively.

Total protein was extracted from Sterivex as described in Hawley *et al.*^40^. Briefly, after thawing Sterivex filters on ice, Bugbuster (Novagen) was added and incubated at room temperature for 20–30 min with rotation. Lysate was removed by extrusion and filters were rinsed with 1 ml lysis buffer. Buffer exchange was carried out on combined lysate using Amicon Ultra 10 K (Millipore) with 100 mM NH_4_HCO_3_ a total of three times with a final volume between 200–500 μl. Protein concentration was determined using the 2-(4-carboxyquinolin-2-yl) quinoline-4-carboxylic acid (Bicinchoninic acid or BCA) assay. Urea was added to a final concentration of 8 M and dithiothreitol added to a final concentration of 5 mM and incubated at 60 °C for 30 min, followed by 10-fold dilution with 100 mM NH_4_HCO_3_. Samples were then subject to trypsin digest at 37 °C for 6 h followed by C18 solid phase extraction and strong cation exchange.

### Environmental DNA and RNA sequencing

Extracted genomic DNA was used to generate small subunit ribosomal RNA (SSU or 16S/18S rRNA) gene pyrotag libraries^20,41^. Pyrotag datasets from HR and LV samples were generated by PCR amplification using universal three-domain forward and reverse bar-coded primers targeting the V6-V8 hypervariable region of the SSU rRNA gene^42^: 926F (5′- AAA CTY AAA KGA ATT GRC GG-3′) and 1392R (5′- ACG GGC GGT GTG TRC-3′). Samples were purified using the QIAquick PCR Purification Kit (Qiagen), and sequenced by 454-pyrosequencing^43^ at the DOE Joint Genome Institute (JGI), or Génome Québec Innovation Centre at McGill University (Table 3 and Supplementary Table 1). iTag datasets from HR and LV samples were generated by PCR amplification using forward and reverse bar-coded primers targeting the V4-V5 hypervariable region of the bacterial and archaeal SSU rRNA gene: 515F (5′- Y GTG YCA GCM GCC GCG GTAA—3′) and 806R (5′- CCG YCA ATT YMT TTR AGT TT -3′)^44,45^. Samples were sequenced according to the standard operating protocol on an Illumina MiSeq platform at the JGI^46^.

Ilumina metagenomic shotgun libraries from LV samples were generated at the JGI and paired end sequenced on the Illumina HiSeq platform^43,47–51^. In addition to the datasets described above, specific methods for full-length SSU rRNA gene, large-insert genomic (fosmid) libraries, Sanger shotgun metagenomes and pyrotags generated from 2006 to 2008 samples have been previously reported^20,27,52^.

Extracted environmental total RNA was used to generate paired end sequenced Illumina metatranscriptome libraries^53^ at the JGI on the HiSeq and (Table 4 and Supplementary Table 2).

### Environmental protein sequencing

Extracted environmental protein was sequenced using tandem mass spectrometry (MS/MS) as described previously^40^. Samples were analysed by capillary liquid chromatography-tandem mass spectrometry (Thermo, LTQ ion trap mass spectrometer or Thermo LTQ-Orbitrap mass spectrometer) in data-dependent mode. Spectra were matched to peptide sequences using the search tool MSGFDBPlus^54^. The amino acid sequence database used for matching spectra to protein sequences was constructed from metagenomic information from Saanich Inlet and the Line P transect in the Northeastern subarctic Pacific Ocean with additional full-length fosmid libraries^20^, and single cell genomes from Saanich Inlet^55^ totalling over 23 million protein sequences.

## Data Records

A Table unifying all multi-omic samples and Saanich Inlet geochemical samples from Torres-Beltrán *et al.*^35^ is summarised in Table 2 and detailed in Supplementary Table 2.

### Small subunit rRNA gene tag sequences

Small subunit rRNA gene pyrotag and iTag sequences are available from the National Center for Biotechnology Information (NCBI) Sequence Read Archive (Data Citation 1). A summary of Pyrotag and iTag samples can be located in Table 1, key to the Pyrotag and iTag data files is located in Table 3 and NCBI BioSample IDs and sequencing centre information for individual samples are located in Supplementary Table 1. In addition, previously published full length small subunit rRNA gene libraries^27^ are archived at GenBank.

### Metagenomes and Metatranscriptomes

Metagenomic and metatranscriptomic data sets are accessible through the JGI IMG/M portal (https://img.jgi.doe.gov/cgi-bin/m/main.cgi) under the study name *Marine microbial communities from expanding oxygen minimum zones in the northeastern subarctic Pacific Ocean* (Data Citation 1). A unifying inventory of metagenomes, metatranscriptomes and metaproteomes sequenced for the Saanich Inlet time-series is summarised in Table 2, with key in Supplementary Table 2 (Data Citation 1). Sanger sequenced fosmid library^28^, 454 and Illumina sequenced metagenomic libraries^20,21^ and 454 sequenced viral metagenomes^33^ have been previously published.

### Metaproteome

Metaproteomic datasets have been deposited in the ProteomeXchange Consortium via the PRIDE^56^ partner repository with the dataset identifier PXD004433 (Data Citation 2). The key detailing the file types available on the PRIDE repository is located in Table 5. Previously published metaproteomic data sets^20^ were archived at *Mass Spectrometry Interactive Virtual Environment*.

## Technical Validation

### Small subunit ribosomal RNA gene tag sequences

For SSU rRNA gene pyrotags and iTags the quality of extracted genomic DNA was verified on 0.8% agarose gels stained with ethidium bromide or SYBR Safe DNA dye (Thermofisher). Samples were run at 16 V overnight with molecular ladders (10 μl of 50 ngml^−1^ of 1 kb+; 2, 5 and 10 μl of 50 ngml^−1^ HindIII ladder) to determine size and estimate concentration of extracted sample DNA (5 μl of extract run on gel). After running, the gel was observed under a UV gel documentation system to check for approximate molecular weight (>36 kb) and evidence of shearing or degradation. In addition, DNA concentration was quantified and corrected to volume filtered using PicoGreen (Thermofisher) following the vendor’s protocol.

**Figure 2:**
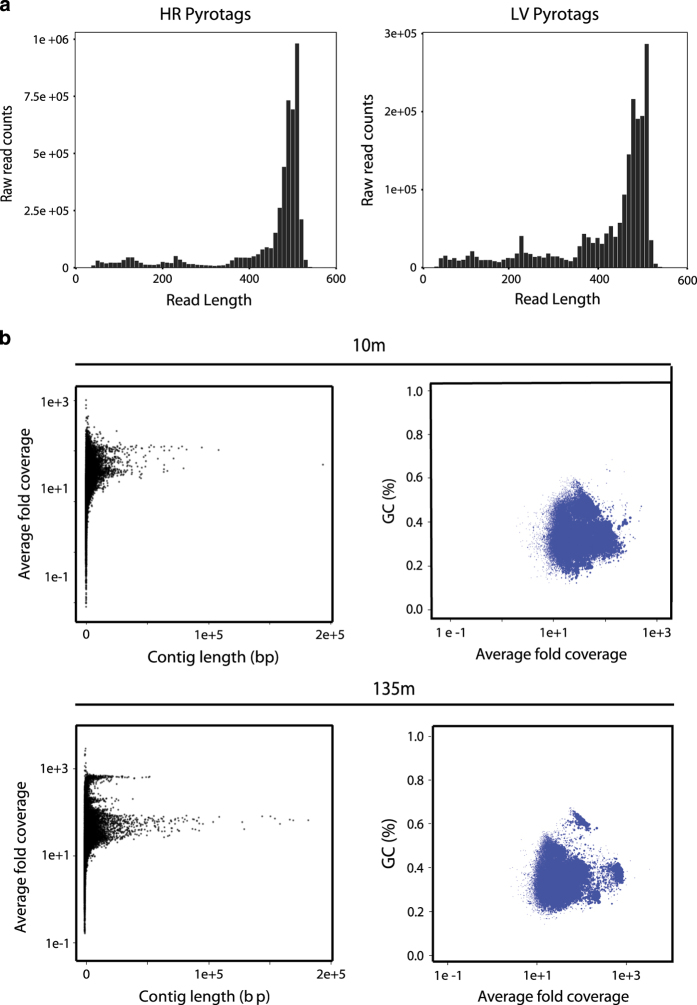
Data Validation figures for SSU rRNA tag sequencing and metagenomes. (**a**) 454 PyroTags for small subunit rRNA gene showing number of raw reads versus read length for large volume samples (99 samples in total) (left) and high resolution samples (311 samples in total) (right). (**b**) Metagenomic assemblies for two samples from different depths showing average fold coverage versus contig length and percentage GC versus average fold coverage for contigs.

Quality control for 454-pyrosequencing entails accurate quantification of prepared library fragments to optimize the sequencing run output, therefore JGI has developed custom qPCR methods to quantify 454 libraries^43^. In addition, an optimized emulsion PCR protocol was developed to significantly improve the coverage in high GC regions that otherwise would be biased^49^. Génome Québec Innovation Centre quality control protocol for 454 pyrosequencing entails the use of the default parameters assigned to the signal processing software for Long Amplicon #3 pipeline as indicated in the Roche ‘454 Sequencing System Software Manual’ V3.0 Section 1.3^53^. For produced 454 pyrotag datasets for both high resolution (HR) and large volume (LV) samples a histogram of raw read counts versus read length (Fig. 2a) was used to determine the success of a run e.g. a majority of reads exceeding 450 base pairs. A plot of read counts versus read length for all HR and LV samples is provided in Fig. 2a.

Quality control protocol for iTag sequencing entails QC amplification with V4-V5 primers prior to sample submission to ensure that samples will be successful in JGI sample prep and that there are no contaminants present that will inhibit amplification. Amplified products are run on agarose gel or Bioanalyzer to ensure sample amplification occurred with the expected band size, ~450 bp for bacterial and archaeal SSU rRNA gene V4-V5 region (including primers). Samples showing proper amplification and sizing are passed for Amplification QC and approved to ship to the JGI for processing. All data from the sequencer were demultiplexed and stored in JGI’s archiving and metadata organizer system (JAMO). Read data was processed through JGI’s centralized rolling quality control system verifying that there were no sequencing issues and removing known contaminant reads using a kmer filter in bbduk. Quality controlled reads were processed by iTagger^45,46^.

### Metagenomic and metatranscriptomic data validation

The quality of DNA used for metagenomic sequencing was determined at the same time as verification for SSU rRNA gene pyrotag and iTag amplification as described above. JGI quality control protocol for metagenomic sequences prior to assembly entails rolling QC, an in-house sequence QC pipeline that performs a set collection of analyses and produces a summary report for each lane of Illumina data produced by the sequencing group. This set of analyses calculates read quality, measures sequence uniqueness, and detects abnormal sequence motifs. An assembly, using Velvet, was used to measure coverage and detect contamination^48^. For individual sample assemblies the average fold coverage versus the contig length (Fig. 2b) was plotted and should have a distinct shape for different samples where peaks in contig length representing at a specific coverage represent a given closely related microbial population. Additionally, the percent GC versus average coverage can be plotted, again with distinct shapes for different samples and clusters representing divergent microbial populations.

**Figure 3:**
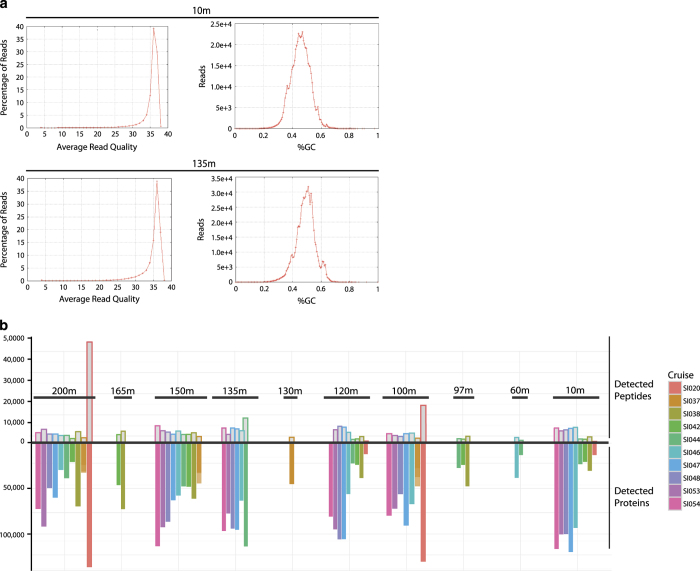
Data validation figures for metatranscriptomes and metaproteomes. (**a**) Metatranscriptomic reads for two samples from different depths showing distribution of reads over read quality (left) and percentage GC (right). (**b**) Metaproteome showing number of detected peptides (top) and detected proteins (bottom) for each depth sampled, colour coded by cruise ID. Higher number of detected proteins than peptides is due the sequence redundancy in the metagenomic database used to identify peptides.

The quality of purified RNA was verified on the Bioanalyzer using a RNA nano Analysis Kit (Agilent Technologies) in order to check on the RNA integrity and sample quantification before cDNA library production and sequencing at JGI. Due to variation inherent in environmental samples the RNA integrity number (RIN) varied between 5.5 and 9 for the sample sand averaged 7.3. JGI quality control protocol for metatranscriptomic sequencing preparation follows the ‘TruSeq Stranded Total RNA Sample Preparation Guide’ (Illumina). Briefly this protocol removed ribosomal rRNA with RiboZero, followed by RNA fragmentation for first strand cDNA synthesis. This was followed by second strand synthesis and subsequent ligation of adapters. After PCR amplification library quality was checked using Bioanalyzer for fragment size (260 bp) and purity. Indexed (barcoded) libraries were normalized to 10 nM and pooled in equal volumes. Transcriptomes were assembled *de novo* and or mapped to a corresponding metagenome. For Additional quality assessment of sequencing run for each sample, histograms of percentage of reads verse average read quality and of reads per percent GC were generated (Fig. 3a). Information on run quality for individual samples is available via the JGI genome portal.

### Metaproteomic data validation

The quality of LC-MS runs were monitored visually by the instrument operator by individually inspecting each analytical run for instrument response, retention time characteristics, and background interference. QC was further monitored using a whole cell digest of *Shewanella oneidensis* that is prepared in bulk and routinely used across all LC-MS systems in the EMSL production labs. Analysis of this QC standard is fully automated once uploaded to an in-house data management system and reports back unique peptide identifications, chromatographic peak width, and mass error within ~1 h. Additionally, these data are subjected to dozens of other analytical metrics that can be further assessed as needed (i.e., when the first pass visual and 1 h results are in question). This QC standard was run at least once per week but generally more often, between sample batches (a single project), and sometimes between sample blocks (when a batch is relatively large and a blocking scheme has been utilized) due to the large diversity of samples analysed in-house. New LC columns were conditioned and tested prior to use on project samples by running a minimum of three QC standards. Blanks were always run between QC standards and between sample batches, but not necessarily between sample blocks. For peptide mapping to full length protein sequences the False Discovery Rate (FDR) was calculated using the spectra to peptide matches that resulted in reversed hits from the on-the-fly reversed database search and a filter on the MSGF value. Number of peptides and proteins detected varies between samples (Fig. 3b). Due to the large size of metagenomic dataset used and redundancy in protein sequences because of multiple sampling of the same environment in the Saanich Inlet time series most peptides mapped to multiple identical proteins, resulting in a greater number of proteins identified then peptides.

## Usage Notes

### Suggested modes of downstream data analysis

In brief we describe the main workflow used in the analysis of SSU rRNA gene pyrotag and iTag datasets. Tag sequences were clustered into operational taxonomic units (OTUs) using the Quantitative Insights Into Microbial Ecology (QIIME) software package^57^ and annotated using the SILVA or GreenGenes databases^58,59^. Community structure was determined using relative abundance and distribution of obtained microbial OTUs along with statistical analyses such as hierarchical clustering and indicator species analysis to identify characteristic groups of OTUs occurring under specific water column conditions such as different water column O_2_ concentrations.

Illumina metagenomes, metatranscriptomes and metaproteomes were analysed using MetaPathways (version 2.0 and 2.5), an open source pipeline for functional annotation and prediction of reactions and pathways in metagenomes and metatranscriptomes^60^. Direct link to software download and specifications can be found online on the Hallam Lab Github repository (https://github.com/hallamlab/metapathways2).

With respect to the metaproteomic datasets there is redundancy in the protein database used for peptide mapping in that amino acid sequences will have different names but identical sequences, and thus a given peptide may map to multiple sequences. To manage this one could cluster the proteins by sequence identity at the amino acid level prior to mapping. In previous publications we have calculated a normalised spectral abundance factor (NSAF) as a pseudo-quantative metric to compare abundance of the same proteins between samples^40^.

## Additional information

**How to cite this article:** Hawley, A. K. *et al.* A compendium of multi-omic sequence information from the Saanich Inlet water column. *Sci. Data* 4:170160 doi: 10.1038/sdata.2017.160 (2017).

**Publisher’s note:** Springer Nature remains neutral with regard to jurisdictional claims in published maps and institutional affiliations.

## Supplementary Material



Supplementary Table S1

Supplementary Table S2

## Figures and Tables

**Table 1:** Summary of datasets, number of samples and sizes for SSU rRNA gene tag sequences.

	**Number of Samples**	**Avg. Number of Raw Reads** *	**Minimum Number of Reads**	**Maximum Number of Reads**
LV PyroTags	99	49,635	286,755	831
LV iTags	19	306,365	1051	355,883
LV_PF iTags	16	345,756	78,925	377,992
HR PyroTag	311	118,641	981,153	2752
HR iTags	47	315,487	1034	448,003

*200–540 bp for PyroTags and >130 bp for iTags.

**Table 2:** Summary of datasets, number of samples and sizes for metagenome, metatranscriptome and metaproteome sequencing.

	**Number of samples**	**Avg. Assembly Size**	**Avg. Scafold Count**	**Average Gene Count**
Metagenomes	90	1.80E+08	3.43E+05	4.69E+05
Metatranscriptomes	62	4.65E+07	1.09E+05	1.22E+05
	**Number of samples**	**Avg. number of Peptides Detected**	**Avg. number of Proteins Detected**	
Metaproteomes	68	4.76E+03	5.81E+04	

**Table 3:** Key to data files in Supplementary Table 1 SSU rRNA gene tag inventory.

**Data field**	**Description**
*Sample ID*	Identifier of unique time-series time point and depth in which seawater sample for dataset was obtained, links to geochemical time series data (Torres Beltrán *et al.*)
*Cruise ID*	Numerical identifier of individual cruises
*Year*	Year of cruise
*Month*	Month of cruise
*Station*	Indicates the sampling station from which seawater sample was obtained
*Depth*	Depth at which seawater sample was obtained
*LV PyroTag*	NCBI BioSample ID for PyrtoTag (V6-V8 region) sequenced pre-filtered samples (2.7–0.22 μm fraction) on NCBI website (http://www.ncbi.nlm.nih.gov/)
*LV iTag*	NCBI BioSample ID for iTag (V4-V5 region) sequenced pre-filtered samples (2.7–0.22 μm fraction) on NCBI website (http://www.ncbi.nlm.nih.gov/)
*LV_PF iTag*	NCBI BioSample ID for PytoTag (V6-V8 region) sequenced pre-filter samples (>2.7 μm fraction) on NCBI (http://www.ncbi.nlm.nih.gov/)
*HR PyroTag*	NCBI BioSample ID for iTag (V6-V8 region) sequenced non-pre-filtered samples (>0.22 μm fraction) on NCBI website (http://www.ncbi.nlm.nih.gov/)
*HR PyroTag Sequencing Centre*	Sequencing centre for HR PyroTag samples. JGI denotes Joing Genome Institute, GQ denotes Genome Quebec. All other sequencing was carried out at JGI.
*HR iTag*	NCBI BioSample ID for iTag (V4-V5 region) sequenced non-pre-filtered samples (>0.22 μm fraction) on NCBI website (http://www.ncbi.nlm.nih.gov/)

**Table 4:** Key to the data fields in the Supplementary Table 2: Metagenomes (MetaG), Metatranscriptomes (MetaT), and Metaproteomes (MetaP) inventory.

**Data field**	**Description**
*Sample ID*	Identifier of unique time-series time point and depth in which seawater sample for dataset was obtained, links to geochemical time series data (Torres Beltrán *et al.*)
*Cruise ID*	Numerical identifier of individual cruises
*Year*	Year of cruise
*Month*	Month of cruise
*Station*	Indicates the sampling station from which seawater sample was obtained
*Depth*	Depth at which seawater sample was obtained
*Tag Data*	Indicates if SSU rRNA tag data exists for that sample and what type of tag (see Table 4)
*MetaG IMG/M Genome ID*	JGI Project ID for the IMG/M website (https://img.jgi.doe.gov/cgi-bin/m/main.cgi) containing metagenome assemblies and annotations
*MetaG BioSample Accession*	NCBI BioSample ID for metatranscriptome at NCBI website (http://www.ncbi.nlm.nih.gov/) with links to sequence read archives (SRA)
*MetaT IMG/M Genome ID*	JGI Project ID for the IMG/M website (https://img.jgi.doe.gov/cgi-bin/m/main.cgi) containing metatranscriptome assemblies and annotations
*MetaT BioProject Accession*	NCBI BioSample ID for metatranscriptome at NCBI website (http://www.ncbi.nlm.nih.gov/) with links to sequence read archives (SRA)
*MetaP Pride File Prefix*	File name prefix in PRIDE database website (https://www.ebi.ac.uk/pride/archive/) for metaproteome samples

**Table 5:** Key to files in PRIDE metaproteome repository PDX004433.

**Data field**	**Description**
*Search Files*	Parameter and settings files used for the database search of spectra to peptide
*Peak Files*	De-isotoped values of mass, observed charged states, and chromatographic elution times from the mass spectrometry runs
*RAW Files*	Mass spectrometry run files, in original format
*FASTA Files*	Amino Acid sequence file for all detected proteins from all Saanich Inlet metaproteome samples.
*SBI_Metagenome2015_AllPeptides*	Tabular lists of identified peptides, associated confidence scores, and protein reference names
*AllProteinsAllExperiments*	Protein lists from all samples, including redundant peptide to protein matches
*Filtered fasta*	FASTA with duplicate sequences removed, cleaned and trimmed for use with the search engine
